# Cytokine and Protein Markers of Leprosy Reactions in Skin and Nerves: Baseline Results for the North Indian INFIR Cohort

**DOI:** 10.1371/journal.pntd.0001327

**Published:** 2011-12-13

**Authors:** Diana N. J. Lockwood, Lavanya Suneetha, Karuna Devi Sagili, Meher Vani Chaduvula, Ismail Mohammed, Wim van Brakel, W. C. Smith, Peter Nicholls, Sujai Suneetha

**Affiliations:** 1 Faculty of Infectious and Tropical Diseases, London School of Hygiene and Tropical Medicine, London, United Kingdom; 2 Blue Peter Research Centre, LEPRA India, Hyderabad, India; 3 Institute of Pathology, Safdarjung Campus Hospital, New Delhi, India; 4 Royal Tropical Institute, Leprosy Unit, Amsterdam, The Netherlands; 5 School of Public Health, University of Aberdeen, Aberdeen, United Kingdom; 6 School of Health Sciences, University of Southampton, Southampton, United Kingdom; University of California San Diego School of Medicine, United States of America

## Abstract

**Background:**

Previous studies investigating the role of cytokines in the pathogenesis of leprosy have either been on only small numbers of patients or have not combined clinical and histological data. The INFIR Cohort study is a prospective study of 303 new multibacillary leprosy patients to identify risk factors for reaction and nerve damage. This study characterised the cellular infiltrate in skin and nerve biopsies using light microscopic and immunohistochemical techniques to identify any association of cytokine markers, nerve and cell markers with leprosy reactions.

**Methodology/Principal Findings:**

TNF-α, TGF-β and iNOS protein in skin and nerve biopsies were detected using monoclonal antibody detection immunohistochemistry techniques in 299 skin biopsies and 68 nerve biopsies taken from patients at recruitment. The tissues were stained with hematoxylin and eosin, modified Fite Faraco, CD68 macrophage cell marker and S100.

**Conclusions/Significance:**

Histological analysis of the biopsies showed that 43% had borderline tuberculoid (BT) leprosy, 27% borderline lepromatous leprosy, 9% lepromatous leprosy, 13% indeterminate leprosy types and 7% had no inflammation. Forty-six percent had histological evidence of a Type 1 Reaction (T1R) and 10% of Erythema Nodosum Leprosum. TNF-α was detected in 78% of skin biopsies (181/232), iNOS in 78% and TGF-β in 94%. All three molecules were detected at higher levels in patients with BT leprosy. TNF-α was localised within macrophages and epithelioid cells in the granuloma, in the epidermis and in dermal nerves in a few cases. TNF-α, iNOS and TGF-β were all significantly associated with T1R (p<0.001). Sixty-eight nerve biopsies were analysed. CD68, TNF-α and iNOS staining were detectable in 88%, 38% and 28% of the biopsies respectively. The three cytokines TNF-α, iNOS and TGF-β detected by immunohistochemistry showed a significant association with the presence of skin reaction. This study is the first to demonstrate an association of iNOS and TGF-β with T1R.

## Author Summary

Leprosy affects skin and peripheral nerves. Although we have effective antibiotics to treat the mycobacterial infection, a key part of the disease process is the accompanying inflammation. This can worsen after starting antibacterial treatment with episodes of immune mediated inflammation, so called ‘reactions’. These reactions are associated with worsening of the nerve damage. We recruited a cohort of 303 newly diagnosed leprosy patients in North India with the aim of understanding and defining the pathological processes better. We took skin and nerve biopsies from patients and examined them to define which molecules and mediators of inflammation were present. We found high levels of the cytokines Tumour Necrosis Factor alpha, Transforming Growth Factor beta and inducible Nitric Oxide Synthase in biopsies from patients with reactions. We also found high levels of bacteria and inflammation in the nerves. These experiments tell us that we need to determine which other molecules are present and to explore ways of switching off the production of these pro-inflammatory molecules.

## Introduction

Leprosy is complicated by leprosy reactions and the development of nerve damage. These are immune mediated events and may occur before diagnosis, during and after anti-bacterial multi drug treatment [1]. Understanding the pathology of these episodes and identifying risk factors for them is important for developing strategies to reduce nerve damage.

The ILEP Nerve Function Impairment in Reactions (INFIR) is a prospective cohort study comprised of 303 new multibacillary (MB) leprosy patients from North India who were recruited to study risk factors for reactions and nerve damage in leprosy [2]. After recruitment they were assessed monthly for a year and then every two months until 24 months. They had detailed clinical and comprehensive neurological examinations and blood was taken at every visit. Skin biopsies were taken from all patients at recruitment and then again if they developed a Type 1 Reaction (T1R). Nerve biopsies were taken when patients had evidence of acute nerve function impairment. Inclusion criteria for the study allowed recruitment of patients with reactions and new nerve damage enabling us to compare patients with and without reactions. Linking clinical, histological and immunological data on patients enables us to test whether particular cytokines or cell types were markers for nerve damage and reactions. It also allowed us to make a comprehensive assessment of the pathology of leprosy lesions as seen in this cohort at baseline. We were also able to compare the histological features of leprosy pathology in skin and nerve. Previous publications from this cohort have reported on the clinical features of nerve damage and clinical markers for reactions [2], [3].

The immunopathology underlying T1R is increased cell mediated immunity with CD4 and macrophage cell activation with production of the Th1-type cytokines interferon-gamma (IFN-γ), interleukin-2 (IL-2) and interleukin-12 (IL-12) [4]. The immunopathology underlying Erythema Nodosum Leprosum (ENL) is that of an antigen-antibody mediated vasculitis [5]. We studied a panel of cytokines and markers that previous small studies have shown to be associated with the pathology of T1R, Tumour Necrosis Factor alpha (TNF-α), inducible Nitric Oxide Synthase (iNOS) and Transforming Growth Factor beta (TGF-β). Macrophages were located using a CD68 marker and S100 staining was used to assess nerve fibre integrity.

The pro-inflammatory cytokine TNF-α is crucial to anti-mycobacterial immunity and plays an important role in granuloma formation during mycobacterial infection [6], [7], [8], [9], [10]. TNF-α protein has been detected in skin lesions across the leprosy spectrum with greater production of TNF-α in lesions from tuberculoid leprosy (TT) patients in whom granuloma formation is better and few or no mycobacteria are detectable. However, excess TNF-α can cause tissue damage in diseases such as rheumatoid arthritis and inflammatory bowel disease, and thus this cytokine plays a key role in tissue damage in leprosy. TNF-α protein has been detected in biopsies taken from leprosy patients with skin reactional lesions, both T1R and ENL [10], [11], [12]. A study in Indian patients found that a higher percentage of cells producing mRNA and protein for TNF-α were found in skin and nerve biopsies with T1R lesions when compared with biopsies from non-reactional borderline tuberculoid (BT) or borderline lepromatous (BL) skin lesions [13], [14]. We therefore hypothesised that high TNF-α production might be a key part of the pathological process of leprosy reactions; then finding high levels of TNF-α in skin biopsies might be a marker for leprosy reactions.

iNOS is an enzyme responsible for synthesis of reactive nitrogen radicals involved in killing of mycobacteria [8]. Expression of iNOS is associated with bacterial killing and a previous study done in India showed high levels in skin lesions of TT and absence in lepromatous leprosy (LL) [15]. High levels of iNOS have also been found in skin biopsies from leprosy patients experiencing T1R in both India and Ethiopia [4], [16]. Increased level of iNOS in T1R are consistent with an immunological model in which patients moving towards the tuberculoid end of the spectrum have increased cell mediated immunity and enhanced intracellular killing capacity of macrophages [15]. A study in Ethiopian patients showed that iNOS was found to be associated with dermal nerves in the granulomas of BL leprosy and might have a role in nerve damage. We therefore hypothesised that a marker for T1R would be increased iNOS in the skin and nerve lesions of patients.

TGF-β is a multifunctional cytokine and, depending on the environment and concentration, has both pro- and anti-inflammatory properties [17], [18]. TGF-β induces wound healing by chemoattraction of monocytes and leukocytes, induction of angiogenesis and control of inflammatory mediators. The anti-inflammatory effects of TGF-β include antagonism of T cell proliferation [19] and IFN-γ, TNF-α and iNOS production [20], [21]. Immunohistochemical studies have showed that active TGF-β1 is present in skin lesions throughout the leprosy spectrum, though in greatest abundance in LL [15]. Accumulation of TGF-β1 in lepromatous lesions may play a role in the absence of iNOS. Levels of active TGF-β1 are low in T1R compared to non-reactional BT/BL skin and hence may be an important factor contributing to the unregulated inflammatory process.

The location and distribution of CD4 and CD8 cells in leprosy lesions have been well described; tuberculoid lesions have abundant T cells in the granuloma of a CD4 lineage, fewer T cells are found in the LL skin lesions and they have a higher proportion of CD8 cells [22]. The CD4 cells in tuberculoid lesions also have a typical location with CD4 cells in the central area and a rim-like mantle of CD8 cells. In lepromatous lesions CD8 cells are admixed with macrophages and CD4 cells [23]. Narayanan *et al* described the distribution of T cells in leprosy reactional lesions [24]. Although macrophages are a key cell in the leprosy granuloma, only one small study has looked at the distribution of macrophages in leprosy reactional skin lesions and high numbers of CD68 cells were found in T1R lesions [13]. There has been no systematic study of the distribution of CD68 cells in lesions across the spectrum and comparing the distribution in reactional and non-reactional skin lesions.

S100 is a specific nerve tissue protein and can be stained for histologically [25]. Several studies have demonstrated and described the utility of S100 staining as an aid to visualise dermal nerves, and to assess their morphology and nerve damage [26]. We added S100 staining to our panel of markers to aid assessing the morphology of nerves.

Previous pathological studies have been done on small groups of patients, often with poorly described case definitions. This large cohort study is the first large field based study to include pathological investigations in the assessments. The adequate sample size allows us to compare the different types of leprosy and to test for the effect of various factors relating to leprosy reactions.

Previous studies reported that the pathology of leprosy in nerve biopsies is often more severe than that of skin lesions, with a heavier bacterial load and more inflammation in nerves. We tested the following hypotheses in relation to identifying cytokines and proteins as markers for reactions and leprosy nerve damage in both skin and nerve biopsies taken in this cohort:

1. TNF-α levels would be higher in reactional lesions
2. iNOS levels would be higher in reactional lesions
3. TGF-β levels would be reduced in reactional lesions
4. Nerve lesions would have a more severe pathology than skin lesions with more inflammation.

## Methods

### Ethical considerations

The study was approved by the ethics committee of the Central JALMA Institute for Leprosy, a major leprosy research centre of the Indian Council for Medical Research. No financial incentives were given to participants. Informed written consent was obtained from individual study subjects before inclusion in the study, using a standard consent form. Further details are available elsewhere [2].

### Design

This was a cohort study of 303 newly registered MB patients. The patients were followed up monthly for one year and every second month during the second year.

### Location

Recruitment of subjects took place in The Leprosy Mission (TLM) hospitals in Naini and Faizabad, specialist leprosy referral centres in Uttar Pradesh, North India. The immunological and histopathological investigations were carried out at the LEPRA Society Blue Peter Research Centre in Hyderabad, Andhra Pradesh and at the TLM Stanley Brown Laboratories formerly located in Miraj, Maharashtra now in Shahdra, New Delhi.

### Study population

The study population comprised newly registered MB patients requiring a full course of multi drug therapy. A detailed description of the study design has already been published [27].

### Definitions

**Nerve function assessment:** Nerve function impairment was present when a patient had either or both motor or sensory loss which were assessed by voluntary muscle testing and with Semmes-Weinstein monofilaments as described previously [2]. Nerve function impairment was categorised as old when signs and symptoms had been present for more than six months and new when signs and symptoms had been present for six months or less.

**Figure 1:**
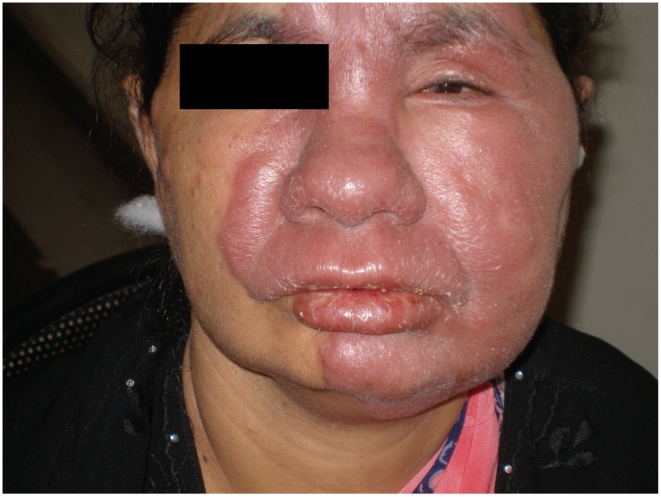
Patient with borderline tuberculoid leprosy face lesion with severe Type 1 Reaction. Facial patch in severe Type1 Reaction with erythema and oedema of the lesion and mild scaling.

**Type 1 or Reversal Reaction:** T1R was diagnosed when a patient had erythema and oedema of skin lesions. This may have been accompanied by neuritis and oedema of the hands, feet and face. A patient could have a skin reaction only, a nerve reaction only, or a skin and nerve reaction. Figure 1 shows a typical skin reaction lesion.

**Erythema Nodosum Leprosum:** ENL was diagnosed when a patient had crops of tender subcutaneous skin lesions. There may have been accompanying neuritis, iritis, arthritis, orchitis, dactylitis, lymph-adenopathy, oedema and fever.

### Database

All the data obtained in the study, including the clinical, neurophysiological, serological and histopathological, were entered on a computer locally and subsequently merged into a single Microsoft Access database. Further details of the study, methods, definitions, documentation and the status of the cohort at baseline have been published [2].

### Skin biopsies

All patients had an elliptical incision skin biopsy taken from an active skin lesion at enrolment.

### Nerve biopsies

These were done when patients had evidence of acute neuritis. A partial thickness biopsy was taken from a palpable regional cutaneous nerve. The biopsies were split in half, and one portion was fixed in 10% buffered formal saline and the other snap frozen in liquid nitrogen. The biopsies were transported in liquid nitrogen or formalin to the Blue Peter Research Centre, Hyderabad for processing and analysis.

### Biopsy processing

The skin and nerve biopsies were processed and embedded in paraffin and serially sectioned in the saggital plane at 5 µ thickness on a Leica microtome. Sections were stained with Haematoxylin and Eosin (H&E) stain to study morphology and modified Fite-Faraco to identify acid fast bacilli. Acid fast bacilli was graded according to Ridley's scale of 0 to 6+ as Bacillary Index of Granuloma.

### Biopsy assessments

The biopsy assessments were done using a standardised set of definitions for histological features and recorded on a proforma. A single pathologist (SS) reviewed the H&E and Fite Faraco stained sections and assessed the diagnosis of leprosy, assigned each case a Ridley-Jopling classification and assessed the presence of leprosy reaction and neuritis.

### Skin biopsy

#### Diagnosis of leprosy

This was confirmed when evidence of nerve inflammation and/or acid fast bacilli were seen and a granulomatous inflammation consistent with leprosy was present [28]. The following morphological features were assessed on all sections:

1. **Cellular infiltrate/granuloma:** The assessment of the cellular infiltrate consisted of identifying granuloma formation, type, population and maturity of the cells making up the granuloma and the fraction of the dermis occupied by the infiltrate (interpreted as granuloma fraction).
2. **Oedema:** This was present in dilated vascular channels (capillaries and lymphatics), causing splaying out of the dermal collagen. Extra cellular oedema was present when wide separation of cellular infiltrate was present and intracellular oedema when ballooning of individual cells was seen.
3. **Nerve inflammation:** This was graded as perineural inflammation when the inflammatory cells were present around the nerve and intraneural inflammation when the inflammatory cells were found inside a nerve.
4. **Necrosis:** This was reported as present when caseous necrotic foci and apoptotic cells were seen in the infiltrate.
5. **Bacterial Index of Granuloma:** This was graded from 0 to 6+ based on Ridley's scale.
6. **Ridley-Jopling Classification:** This was used in the diagnosis of leprosy patients.

#### Lepromatous leprosy (LL)

When macrophage and foam cell collections present with numerous bacilli interspersed with sparse number of lymphocytes.

#### Borderline lepromatous (BL) leprosy

When there were macrophage granulomas, numerous lymphocytes and moderate numbers of bacilli.

#### Borderline tuberculoid (BT) leprosy

When lympho-epithelioid granuloma presents with occasional Langhans giant cells.

#### Tuberculoid leprosy (TT)

When immature epithelioid cells are present together with Langhans giant cells and numerous lymphocytes.

#### Indeterminate leprosy

When some nerve inflammation is seen with rare acid fast bacilli and an absence of clear epithelioid or macrophage granulomas.

#### Non-specific inflammation

Used as a diagnostic category when inflammation was present without the specific features for leprosy, notably neural inflammation and acid fast bacilli.

#### Type 1 Reaction (T1R)

When at least two of the following features were present: granulomas with extra and intracellular oedema, dilated vascular channels, separation of dermal collagen, evidence of an intense delayed-type hypersensitivity (DTH) response with acute damage to dermal nerves and granuloma [29].

#### ENL reaction

When a polymorphonuclear neutrophilic infiltrate on the background of a macrophage granuloma accompanied by oedema and often with evidence of vasculitis and/or panniculitis was seen [30].

### Nerve biopsy

Nerve biopsy sections were reviewed using the same criteria and graded into Ridley-Jopling classes.

#### Borderline tuberculoid (BT) leprosy

When the nerve was infiltrated by multiple collections of epithelioid cells, giant cells and focal aggregates of lymphocytes.

#### Borderline lepromatous (BL) leprosy

When there were sheets of macrophages with acid fast bacilli interspersed with lymphocytes and a few plasma cells.

#### Lepromatous leprosy (LL)

When numerous bacilli in Schwann cells and relatively few intraneural inflammatory cells were present.

#### Indeterminate leprosy

When a mild lymphohistiocytic infiltrate with a rare acid fast bacilli was present.

#### Neuritis and T1R in nerve

When there was oedema in the nerve with wide separation of the axons and an increased population of inflammatory cells.

### Validation of histological diagnosis

A purposely selected sample of 68 slides was sent to a second pathologist who used the same scoring system for diagnosis of leprosy and reactions. The selection covered the full range of leprosy types and reactions. The paired assessments made by SS and by an independent assessor blinded to the assessment by SS were compared. There was perfect or good agreement on the Ridley-Jopling classification in all 51 biopsies (this excludes biopsies that showed non-specific inflammation). For the bacterial index assessment the Kappa was 0.5 and for the granuloma fraction assessment the kappa was 0.6 indicating good agreement. SS diagnosed T1R in 20/66 biopsies and MJ in 11/66. There were four T1R diagnosed by MJ but not SS and 13 diagnosed by SS but not MJ. Comparing the ENL diagnoses showed that SS diagnosed 6/66 as having ENL and MJ 4/66. They only agreed on one ENL diagnosis.

### Immunohistochemical staining

Sections were mounted on Poly-L-Lysine (BDH, Poole, UK) coated slides. They were stained using the following series of incubations and washes: xylene - 10 minutes, two changes; 100% ethanol - 5 minutes, two changes; 70% ethanol - 5 minutes and 50% ethanol - 5 minutes. Endogenous peroxidase activity was quenched with 0.06% hydrogen peroxide in absolute methanol for 10 minutes and then rinsed in distilled water. The sections were incubated with trypsin at a concentration of 1 mg/ml for 30 minutes at 37°C in 0.1 mM CaCl_2_. Monoclonal antibodies to TNF-α (Monosan 5006, Monosan), CD68 (M0876, DAKO, Denmark) and iNOS (610328, BD Transduction Laboratories, USA) and polyclonal antibodies to S100 (Z0311, DAKO, Denmark) and TGF-α (sc-146, Santa Cruz Biotechnology Inc., USA) were used to detect epitope immunoreactivity in paraffin sections of skin and nerve biopsies of leprosy patients. Standardisation of incubation times and antibody concentrations was carried out.

The immunohistochemical technique involved the sequential application of the following reagents in a humidified chamber: blocking with normal rabbit sera 1∶10 (for monoclonal antibodies) and normal swine sera 1∶10 (for polyclonal antibodies) in phosphate buffered saline for 30 minutes followed by incubation with the monoclonal antibodies CD68, TNF-α, iNOS and polyclonal antibodies TGF-α and S-100 at optimized concentrations in phosphate buffered saline containing 1% bovine serum albumen, for 1 hour at room temperature. A secondary biotinylated rabbit/swine anti-antibody diluted in blocking buffer (1∶250) was interacted with the sections for 30 minutes. This step was followed by incubation with Avidin Biotin complexed horse radish peroxidase (K 0377, DAKO, Denmark) for 30 minutes. Each incubation step was followed by washing the tissue sections in phosphate buffered saline twice (5 minutes each). The controls for the specificity of staining included using normal serum, omitting the primary antibody, and using similar isotype antibodies.

The sites of immunocomplex were identified by light microscopy following treatment with a chromogen, 3′ diamino benzidine tetrachloride. Further, the sections were counter-stained with Harris' haematoxylin for 2 minutes, mounted in DPX mountant and were visualized by an Olympus BX.50 microscope. The Olympus PM-30 exposure control unit was used for microphotography.

The immunohistochemical stained sections were assessed and graded by a pathologist (SS) and three investigators (LS, KS, MC) with regular crosschecking of the process.

The cell and cytokine staining was assessed by grading the sections with the following scale: 0, negative; 1+, a few scattered positive cells; 2+, 10–30% of the cells were positively stained; 3+, 30–50% of the cells were positively stained; 4+, 50–80% of the cells were positively stained; and 5+, 80–100% of the cells were positively stained. Cellular infiltration was assessed with the following scale: 0+, no cellular infiltrate; 1+, groups of cells; 2+, moderate cellular infiltration; and 3+, extensive cellular infiltration. This scale has been used in previous work [18], [20], [23]. For a small number of biopsies the full set of assessments was not completed. This accounts for the variability in numbers in some tables.

### Statistical analysis

To assess the association between these staining groups and variables describing reaction status or Ridley-Jopling classification we used Pearson's chi-squared test.

#### Patients at risk of developing T1R

A sub group of patients at risk of developing T1R was identified. This excluded patients with ENL and the patients with no significant lesions (NSL). Patients with ENL were excluded because they were very unlikely to have T1R and ENL simultaneously at baseline. Patients with NSL were excluded in this analysis because the comparison involved a histological comparison and their biopsy showed so little inflammation that assessing the histological features of reaction was not possible.

## Results

Two hundred and ninety-nine skin biopsies were available for morphological and immunohistochemical assessment. Table 1 gives the Ridley-Jopling classification of skin biopsies by histological assessment. One hundred and twenty-eight biopsies were diagnosed as showing BT; 82 as BL; 28 as LL; 22 as NSL; and 39 as indeterminate.

**Table 1:** Ridley-Jopling classification, Bacterial Index and Granuloma Fraction for skin biopsies.

		Bacterial Index		Granuloma Fraction	
R-J classification	n (%)	Mean	SD	Mean	SD
Borderline tuberculoid (BT)	128 (42.6)	.2	.4	25.8	14.0.
Borderline lepromatous (BL)	82 (27.5)	2.8	1.6	22.9	13.0.
Lepromatous leprosy (LL)	28 (9.4)	4.2	1.3	27.9	18.9
Indeterminate leprosy	39 (13.1)	0	0	7.3	2.8
No significant lesions (NSL)	22 (7.4)	0	0	5.7	2.3
**Total**	299	1.2	1.8		

**Figure 2:**
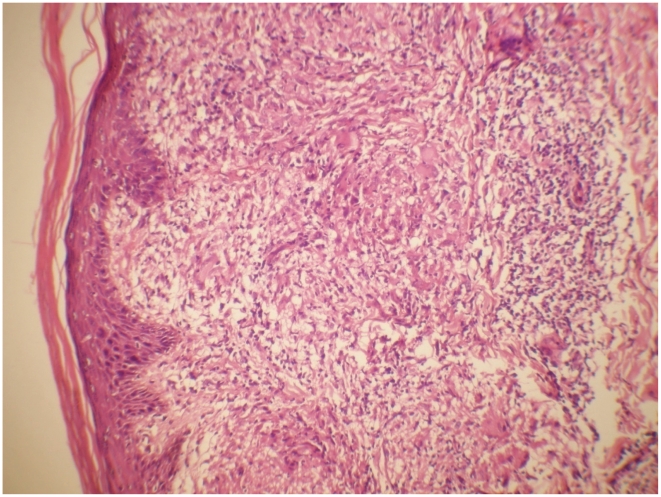
Skin borderline tuberculoid leprosy in Type 1 Reaction. Skin - BT in Type 1 Reaction showing aggressive epithelioid granuloma, epidermal erosion, extracellular oedema and lymphocytic influx. H&E staining ×20.

**Figure 3:**
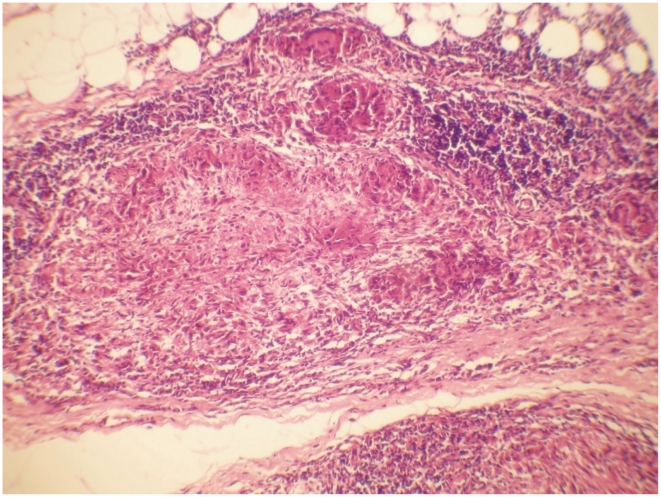
Delayed-type hypersensitivity in dermal nerve. Skin - BT in Type 1 Reaction showing aggressive DTH with epithelioid granuloma destroying a deep dermal nerve, areas of necrosis and focal lymphocytic response. H&E staining ×20.

BT and BL groups were then further classified by the presence or absence histologically of T1R giving 61 BT and 67 BT T1R biopsies and 47 BL and 33 BL T1R biopsies (Table 2). This total of 208 biopsies excludes two BL cases with histological ENL. Among these the biopsies classified as showing evidence of a T1R all had typical features of T1R with prominent oedema and other changes (Figures 2 and 3).

**Table 2:** Classification of patients by Ridley-Jopling and reaction types.

Ridley-Jopling type	N (%)	Reaction categories	
Borderline tuberculoid (BT)	128 (53.6)	BT	61(48%)
		BT in T1R*	67 (52%)
Borderline lepromatous (BL)	82 (34.6)	BL	47 (57.3%)
		BL in T1R	33 (40.2%)
		BL in ENL**	2 (2.4%)
Lepromatous leprosy (LL)	28 (11.8)	LL	20 (71.4%)
		LL in ENL	8 (28.6%)
**Totals**	**238**		**238**

*T1R: Type 1 Reactions.

**ENL: Erythema Nodosum Leprosum.

There were 28 LL patients of whom eight had evidence of ENL. Two BL patients with biopsy evidence of ENL were also added to this group to give a total of 10 biopsies in the ENL group.

The biopsies in this cohort were assigned to different groups for the purposes of analysis and this grouping was used consistently throughout the analysis. The indeterminate/NSL group were considered separately from the main Ridley-Jopling groups. The main comparisons for association of the presence of T1R in the skin biopsy were done on the combined BT and BL group.

### Morphological analysis

Many of the BT case biopsies showed pronounced inflammation (Figures 2 and 3). In the biopsies showing features of T1R, dilated vascular channels, wide separation and paleness of the collagen connective tissue and intra- and extracellular oedema were present as the key histological features. The cellular infiltrate in these biopsies diagnosed with T1R included Langhans and multinucleate giant cells together with lymphocytes. There was a strong, significant association between granuloma fraction and T1R (p<0.01).

### Immunostaining

The level of immunostaining for CD68, TNF-α, TGF-β and iNOS in the different leprosy types and by reaction status are summarised in Table 3.

**Table 3:** Immuno-staining in skin biopsies for CD68, TNF-α, TGF-β and iNOS.*

		BT**	BL***	LL†	No RR‡	RR	ENL§
Level of Staining∥		% (N = 128)	% (N = 82)	% (N = 28)	% (N = 152)	% (N = 113)	% (N = 13)
**CD68**	0	4.7	4.9	3.6	8.6	4.4	7.7
	1	25.0	30.5	10.7	43.4	14.2	15.4
	2	49.2	45.1	57.0	39.5	50.4	53.8
	3	19.5	14.6	21.4	7.2	26.6	15.4
	4	1.6	4.9	7.1	1.3	4.4	7.7
**TNF-α**	0	17.3	28.8	26.9	36.9	12.6	7.7
	1	42.5	31.3	38.5	43.0	28.8	38.4
	2	26.8	26.3	26.9	16.1	36.9	46.2
	3	6.3	13.8	7.7	4.0	13.5	7.7
	4	7.1	0.0	0.0	0.0	8.1	0.0
**TGF-β**	0	6.3	6.1	0.0	9.9	4.5	0.0
	1	20.5	32.9	25.0	47.4	8.0	30.7
	2	39.4	40.2	25.0	31.6	42.9	15.4
	3	26.0	15.9	39.3	9.2	33.0	46.2
	4	7.9	4.9	10.7	2.0	11.6	7.7
**iNOS**	0	21.9	23.5	14.3	31.1	16.8	0.0
	1	27.3	35.8	32.1	37.8	25.7	46.2
	2	38.3	37.0	53.6	29.8	42.5	53.8
	3	12.5	3.7	0.0	1.3	15.0	0.0
	4	0.0	0.0	0.0	0.0	0.0	0.0

*Figures are rounded to 1 decimal place.

**Borderline tuberculoid leprosy;

***Borderline lepromatous leprosy;

**†:** Lepromatous leprosy;

**‡:** Reversal reaction.

**§:** Erythema Nodosum Leprosum;

**∥:** The following molecules were assessed as marker for TIR in skin biopsies: CD68+ (sensitivity 82%, specificity 52%), TNF-α (sensitivity 59%, specificity 80%), iNOS (sensitivity 58%, specificity 69%) and TGF-β (sensitivity 88%, specificity 57%).

#### CD68

**Figure 4:**
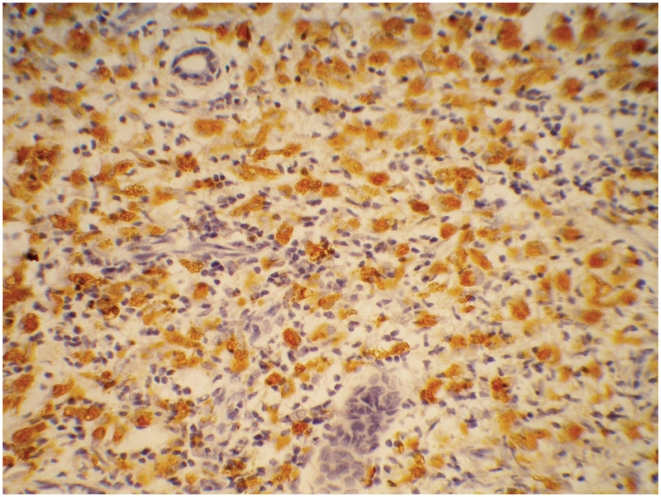
Skin showing CD68 cells. Skin showing CD68 positive macrophages. IHC staining ×40.

CD68+ cells were detected in 95% skin biopsies (Figure 4) from 210 BT and BL cases judged to be at risk of reverse reactions at baseline (Table 3). Cells were located in granulomas (95% biopsies) and dermis (45–59% biopsies). There were low levels of CD68+ cells in the biopsies from the indeterminate/ NSL group with 64% of this group showing grade 1 and 11% grade 2. In this group the cells were located in the dermis and granuloma and none in the dermal nerves. CD68+ staining was significantly higher in biopsies from patients with T1R status (p<0.001) and this association was only present for the BT patients (p<0.001). BL patients had similar levels of CD68+ cells whether or not they were in reaction. CD68+ cells were found principally in the reactional granuloma (95%) and some in the dermis (40%) and the reactional biopsies had significantly fewer CD68+ cells than the non-reactional biopsies (p<0.001). Almost no CD68+ cells were found in the epidermis or dermal nerves for both reactional and non-reactional cases. Nearly all biopsies in the LL group had some CD68+ cells present and these levels were not altered by the presence of ENL. The CD68+ cells were found in the infiltrate and not in the epidermis in both cases and controls with ENL.

#### TNF-α

TNF-α protein was detected in 78% of skin biopsies taken from definite leprosy cases at baseline (181/233). Across the spectrum the BT, BL and LL cases had similar grades of staining with 35–41% of biopsies having staining at grade 2–4. However, only the BT cases had grade 4 staining with 7% cases staining at grade 4; no BL or LL cases stained at grade 4. The TNF-α was located in macrophages and epithelioid cells within the granuloma with no staining in the epidermis, dermis and only 7 cases with staining detectable in the dermal nerves. Comparing the numbers of patients by Ridley-Jopling category and the presence of TNF-α in the macrophages a clear trend was seen with 81% of BT macrophages staining for TNF-α compared with 69% BL and 64% LL. Very little TNF-α was detected in the biopsies of the indeterminate/NSL group with 55% of the group showing staining at grade 0, 30% at grade 1, 15% at grade 2 and 2% at grade 3. This staining was present within the granuloma. Detectable TNF-α in biopsies was significantly associated with T1R (p<0.000) and the levels were similar in both BT T1R and BL T1R patients. There was significantly more TNF-α in both macrophages (p = 0.003) and granulomas (p≤0.001) in biopsies classed as being in T1R. TNF-α was not found in the dermis or the dermal nerves in association with T1R. Using the presence of TNF-α in the skin as a marker for reaction has a sensitivity of 21% and a specificity of 96%. All the cases diagnosed histologically with ENL had some staining for TNF-α, although the levels were quite modest with six cases showing staining at only grades 1 and 3 and one case at grades 2 and 3 respectively. The levels of TNF-α were similar for ENL and non-ENL cases. The biopsies from patients with ENL had 92% staining within the granuloma whilst those from non-ENL cases had 53% (p = 0.015). Patients with ENL had almost significantly higher (p = 0.052) amounts of TNF-α in their macrophages. No TNF-α was detected in the epidermis or dermal nerves.

#### iNOS

**Figure 5:**
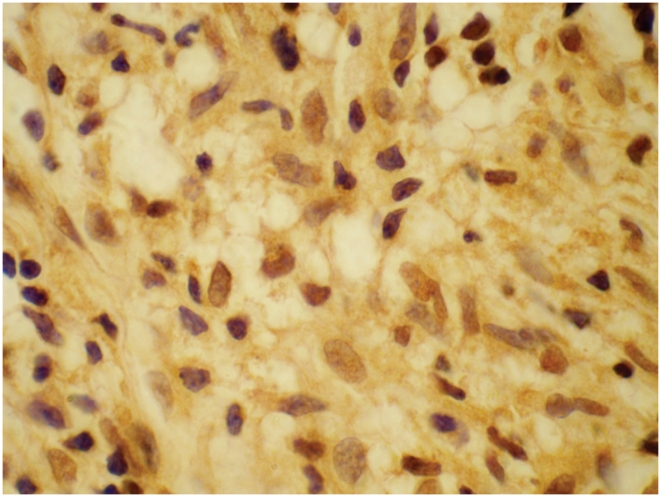
Skin stained for TGF-β. Skin showing TGF-β positive macrophages. IHC staining ×100.

iNOS was detected in 78% (185/237) of skin biopsies from established leprosy cases at baseline (Figure 5). The grade of staining in biopsies from patients across the spectrum was similar. Twelve percent of BT cases stained at level 3 compared with 4% for BL and none for LL suggesting that this molecule is associated with a well expressed cell mediated immunity response. iNOS was detected in several locations - in the dermis (22.4%), granuloma (43%) and sub-epidermal zone (28.6%). iNOS was found in macrophages and epithelioid cells. Comparing iNOS levels in the dermis and granuloma across the Ridley-Jopling spectrum shows a clear trend with highest levels in the dermis and granuloma of BT patients and lowest levels in the LL patients (skin iNOS dermis BT 47.7%, BL 36.4% and LL 15.9%; skin iNOS granuloma BT 45.3%, BL 39.0% and LL 32.1%; p<0.05). This is in accordance with the role of iNOS as a mycobacterial killing agent. The levels of staining for iNOS in the indeterminate group were very similar to those found for TNF-α. Thirty-three percent of indeterminate biopsies had iNOS staining in the dermis with only 12% showing staining in the granuloma.

Significantly more iNOS was detected in biopsies from patients with T1R (73% vs. 83%, p<0.001) and the grade of staining for BT and BL in T1R patients was similar. There was significantly more iNOS in the granulomas of patients in T1R (p≤0.001). iNOS was relatively absent from the dermis with only 15–20% staining positive for iNOS, and absent from the epidermis and dermal nerves. Macrophage staining levels of reactional skin biopsies were non-significantly higher than non-reactional (79.65% vs. 68.4%, not significant).

#### ENL

All patients diagnosed with ENL histologically had some iNOS staining but the differences between ENL and non-ENL patients were not significant. In the ENL group there was some staining for iNOS in the dermis (28%) and some in the granuloma (35%) but none in the epidermis or dermal nerves. One hundred percent iNOS staining was observed in the macrophages of ENL patients.

#### TGF-β

TGF-β was detected in 94% skin biopsies from established leprosy cases at baseline (224/237; Figure 5). All types of leprosy had low to high staining, but biopsies from BT patients had significantly more TGF-β staining than from BL patients (p = 0.05). TGF-β was found almost exclusively in macrophages and epithelioid cells located in granulomas (92–100% biopsies) and dermal nerve (7–12% biopsies). There were low levels of TGF-β staining in the indeterminate/NSL group with 57% of this group having staining at grade 1 and 14% at grade 2. In this group the staining was located in the granuloma with none in the dermal nerves.

#### T1R

TGF-β staining was significantly higher in reactional skin biopsies (reactional vs. non reactional; p<0.001) and this association was stronger for the BT patients (p<0.001) than the BL patients (p = 0.01). TGF-β was located principally in the reactional granuloma but there was no difference between reactional and non-reactional cases in the amount of TGF-β in the dermis or granuloma or the location. Significantly more TGF-β was found in the dermal nerves of cases with T1R (p<0.001). Using the presence of TGF-β in the skin as a marker for reaction has a sensitivity of 44% and a specificity of 88%. All biopsies in the LL group had some staining for TGF-β and there was no difference between patients with or without ENL. No differences were found for cell type or location of TGF-β between cases with or without ENL.

#### S100

S100 staining corresponded inversely with the degree of inflammation, with sections classified as NSL showing a higher degree of staining and active BT and BL sections showing 0 or 1. S100 stain in indeterminate lesions was helpful in localising the nerve and the perineural cellular infiltrate. An active DTH in reactional lesions was accompanied by nerve destruction and consequent absence of S100 staining.

### Nerve biopsies

Table 4 shows the Ridley-Jopling classification for 68 nerves. When the skin and nerve classifications were compared we found that in the three main groups, BT, BL and LL, there was 62%, 66% and 60% agreement between the skin and nerve classification respectively. For the patients with a skin BT classification (n = 29) their nerve biopsies showed two nerves classified as TT, five as BL and one as indeterminate. The BL skin classification (n = 27) showed two nerves classified as BT and four as LL. The skin LL group had two nerves as BL classification. Table 5 shows the comparison between the bacterial index in skin and nerve and that the bacterial index was consistently higher in nerve than skin biopsies. These data suggest that patients are less able to control the mycobacterial infection in their nerves. Oedema was a notable feature in nerves with a reactional pathology and wide separation of axons was present with inflammatory cells and an increased population of lymphocytes as focal collections or scattered in among the inflammatory cells. In the nerves classified as TT T1R, the epithelioid granuloma contained some Langhans giant cells and the lymphocytes were present as a thick cuff around the granulomas. Some nerves were clean with no evidence of intra or perineural inflammation or acid fast bacilli and these were classified as NSL.

**Table 4:** Histological classification of nerve biopsies.

	Bacterial Index		
Classification	N	Mean	SD
Tuberculoid leprosy (TT) in T1R*	2	0	0
Borderline tuberculoid (BT) leprosy	1	1.45	1.2
Borderline tuberculoid (BT) in T1R	20		
Borderline lepromatous (BL) leprosy	6	3.5	1.5
Borderline lepromatous (BL) in T1R	21		
Lepromatous leprosy (LL) / ENL**	7	4.4	.78
Indeterminate leprosy	2	0	0
No significant lesions (NSL)	9	0	0
**Total**	**68**		

*T1R: Type 1 Reactions.

**ENL: Erythema Nodosum Leprosum.

**Table 5:** Comparison of Bacterial Index in skin and nerve biopsies.

	Bacterial Index in nerve biopsy			
Bacterial Index in skin biopsy	0	1–3	4–6	Total
**0**	16	10	4	**30**
**1–3**	3	11	9	**23**
**4–6**	1	2	10	**13**
**Total**	**20**	**23**	**23**	**66**

BT and BL nerve biopsies with evidence of reactional pathology dominate this group with only one BT nerve not showing evidence of T1R. There were also two TT nerves with evidence of reactional pathology. Two nerves showed evidence of indeterminate leprosy and nine nerves had no evidence of inflammation.

The bacterial index for each nerve group is given in Table 4 and shows a higher bacterial index for each group in the nerve than in the skin. Since there was only one nerve with evidence of ENL pathology, comparison of the ENL versus non-ENL pathologies could not be done. The nerve with ENL pathology showed modest level of CD68 staining; iNOS staining at 1, TGF-β staining at 2 and no detectable TNF-α. The comparison for nerves with and without T1R were all based on the BL group (six not in T1R, 21 with T1R).

#### CD68

**Figure 6:**
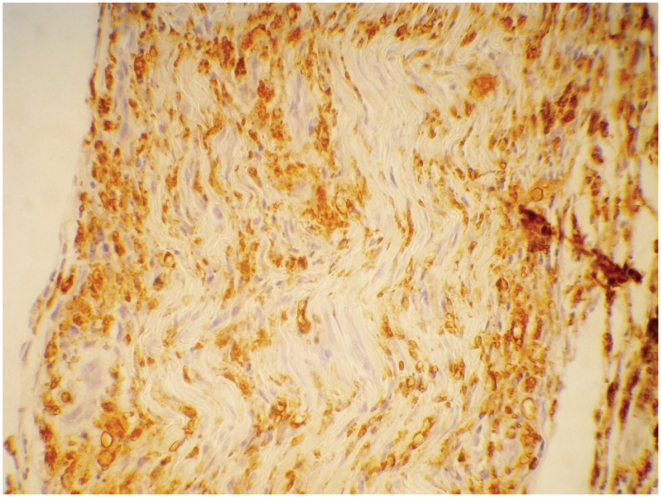
Nerve stained for CD68 cells. Nerve showing CD68 positive macrophages. IHC staining ×40.

Table 6 shows the level of staining for the immuno-markers by leprosy type and reaction status in the nerve biopsies. CD68 staining was detectable in 88% of nerve biopsies at baseline (Figure 6). Higher staining levels were noted in the BL group with 33% of these biopsies staining at grade 3. Zero percent staining for CD68 was located in the granuloma. Significantly higher numbers of CD68 cells were present in nerves with T1R.

**Table 6:** Immuno-staining in nerve biopsies for CD68, TNF-α, TGF-β and iNOS.*

		BT**	BL***	LL†	No RR‡	RR
Level of Staining		% (N = 21)	% (N = 27)	% (N = 7)	% (N = 15)	% (N = 43)
**CD68**	0	10	15	0	20	9
	1	38	26	57	53	30
	2	38	26	29	20	33
	3	14	26	0	0	23
	4	0	7	14	7	5
**TNF-α**	0	38	15	43	27	28
	1	24	41	14	40	30
	2	19	26	14	13	23
	3	19	11	29	20	14
	4	0	7	0	0	5
**TGF-β**	0	11	26	14	33	15
	1	21	22	43	40	24
	2	37	37	43	27	37
	3	21	11	0	0	17
	4	11	4	0	0	7
**iNOS**	0	24	26	0	27	28
	1	48	41	71	40	44
	2	29	30	29	33	26
	3	0	4	0	0	2
	4	0	0	0	0	0

*Percentages rounded to nearest integer because of small numbers.

**Borderline tuberculoid leprosy;

***Borderline lepromatous leprosy;

**†:** Lepromatous leprosy;

**‡:** Reversal reaction.

#### TNF-α

**Figure 7:**
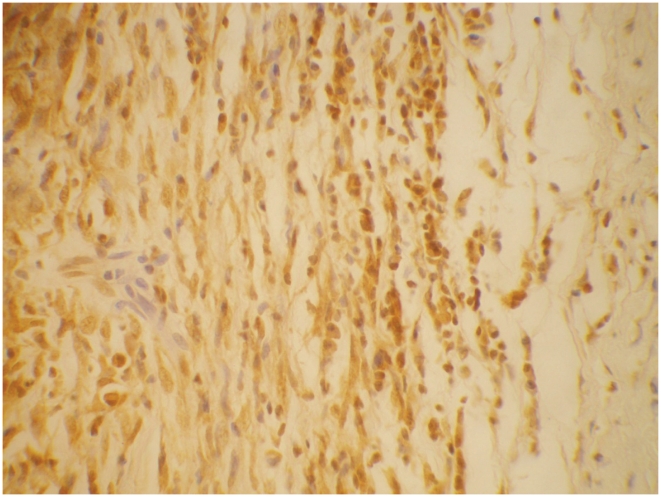
Nerve stained for TNF-α. Nerve showing TNF-α positive macrophages. ICH staining ×40.

TNF-α was detected in 74% of all nerves with 62% of BT in T1R (Figure 7). TNF-α was located in the granuloma and nerves and almost exclusively found in macrophages. In the lepromatous nerves TNF-α was found at grades 0–3 in macrophages.

#### iNOS

There was a variable staining pattern for iNOS in nerves with 28% of TT and no staining in BT and BL. iNOS staining was distributed almost equally between the nerve and the granuloma and associated mainly with macrophages with some staining associated with Schwann cells and axons.

#### TGF-β

**Figure 8:**
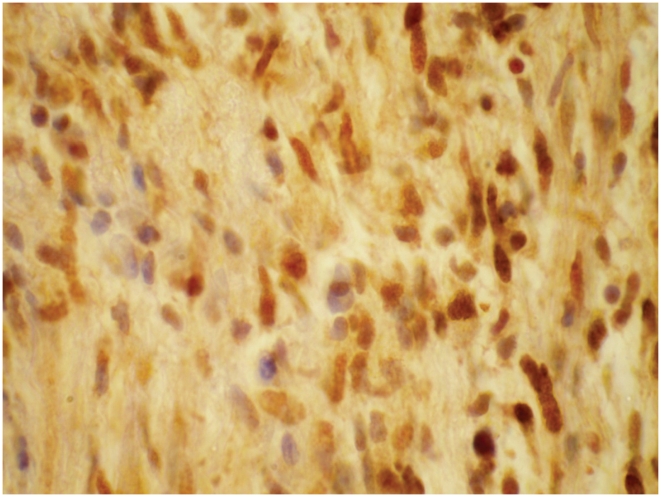
Nerve stained for TGF-β. Nerve showing TGF-β positive macrophages. IHC staining ×100.

TGF-β staining was found across the spectrum of leprosy in nerves and was present at a higher level in BT nerves (Figure 8). It was located in the granuloma associated almost entirely with macrophages. TGF-β staining was not associated with the presence of T1R.

#### S100

**Figure 9:**
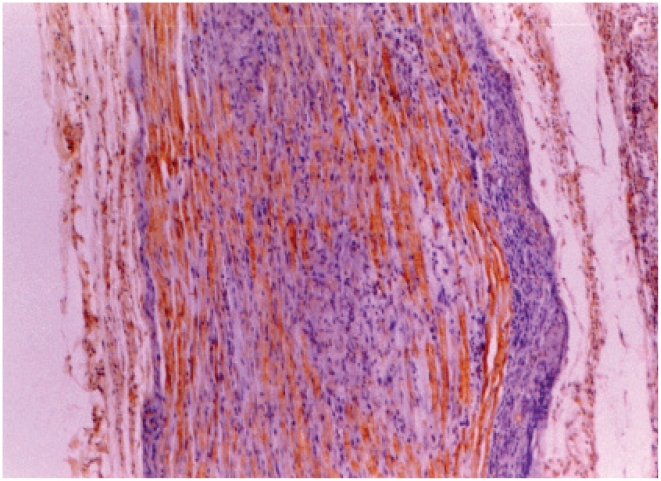
Nerve stained with S100. Nerve showing S100 positive areas of staining of intact fibers and areas of absence of stain in segments of nerve destruction. IHC staining ×20.

S100 staining is a marker of axonal integrity (Figure 9). The level of S100 staining was higher in the NSL group than the indeterminate group reflecting the normality of the nerve structure in the NSL group. Forty-five percent of BT in T1R nerves had a score of 0 or 1 on S100 staining which correlates with the destructive process that is seen in these nerves.

The association between cytokine and marker staining levels (2–4) in skin and nerve was compared (McNemar test) and levels of CD68+ (p<0.01), TGF-β (p = 0.05) and iNOS (p<0.045) were significantly associated, but not for TGF-β.

## Discussion

The examination and comparison of clinical, histological and immunological profiles in a new cohort of newly diagnosed leprosy patients has yielded valuable insights and helped in identification of potential cytokine markers for T1R. This study is the largest prospective study that has combined clinical, histological and immunological investigations on leprosy reactions in a typical leprosy hospital in North India. Although all the patients recruited had multiple leprosy skin and nerve lesions and fulfilled field criteria for MB leprosy, on histological examination the group comprised 42.6% BT, 27.5% BL, 9.4% LL and 13% indeterminate. This indicates that the World Health Organization MB classification is very heterogeneous comprising at least four different Ridley-Jopling types. Of the BT patients, 80% had no mycobacteria detectable either on slit skin smears or in their biopsies. Only 5% of this cohort had highly positive skin smears with a bacterial index of more than 4+. This Indian cohort was dominated by bacteriologically negative but immunologically active BT cases. This was borne out by the high rate of reactions in this cohort with 52% of the BT patients having T1R. The histological findings were typical for the various Ridley-Jopling groups although the bacterial index was lower than anticipated. We also demonstrated histological and immunological differences between the various Ridley-Jopling groups, so confirming that the Ridley-Jopling classification still continues to be useful, especially when the immunological aspects of leprosy are being investigated [31]. A recent study of the histological diagnosis of T1R showed that oedema was one of the most important features in the diagnosis of reactions [29]. This finding was also present here in both the skin and the nerve biopsies. The ENL reactions were also typical with polymorphonuclear leukocyte infiltrate and oedema, often accompanied by vasculitis and panniculitis.

We have demonstrated significant new associations between T1R and the presence of several cytokines. Levels of TNF-α, TGF-β and iNOS when detected by immunohistochemistry were all significantly increased in the skin T1R lesions. A similar pattern of raised cytokine production was found in nerve T1R lesions with the exception of iNOS. In both skin and nerves there were raised numbers of macrophages as assessed by the CD68 marker. This is the first time that iNOS and TGF-β have been found in skin T1R lesions. The strong association of T1R and TGF-β in skin lesions was not predicted since this molecule has both down- and up-regulatory effects.

Previous work has shown that TNF-α is up-regulated in skin and nerve biopsies in patients with T1R and this was confirmed in this cohort [14]. We have also demonstrated that serum TNF levels rise four to eight weeks before a T1R or nerve impairment episode. These findings highlight the key role of TNF-α in leprosy immunopathology. Future studies could look at the role of TNF in inflammatory damage. It would also be interesting to quantify TNF RNA levels and determine when they rise in relation to leprosy reactions.

Our findings in relation to iNOS also complement earlier work showing high levels of iNOS in tuberculoid and borderline patients with T1R [16]. The biopsies in this cohort also show a clear trend with high levels of iNOS in BT patients and very low, especially in the dermis, in LL patients. It was interesting that 33% of the patients with indeterminate leprosy had detectable iNOS in their skin biopsy. This indicates that mycobacterial killing is going on in this group as part of the evolving pathology even though the granulomas are very small.

The levels of TGF-β were surprisingly high across the spectrum. The staining levels for TGF-β were also higher than that found for iNOS in the indeterminate group patients. The strong association of TGF-β with dermal nerves was unexpected. TGF-β has a role in controlling inflammation and maybe this is a neuro-protective response to protect the dermal nerves from the intense inflammation that accompanies leprosy.

The cytokine staining in the ENL biopsies did not show any noteworthy patterns. It was surprising that higher levels of TNF-α were not detected in the ENL lesions. However there were only a few patients with ENL and some of these biopsies showed only mild ENL histologically. The pathology of ENL requires investigation with larger numbers of patients with better clinical and pathological correlation. It is interesting that iNOS was detected in all the biopsies with ENL. This may also be a marker for mycobacterial killing.

It is striking that all the nerve biopsies showed evidence of acute leprosy inflammation with oedema. There was good correlation between the clinical diagnosis of acute nerve function loss and pathological evidence of inflammation. This acute leprosy inflammation was seen in biopsies from patients with all types of Ridley-Jopling classification. This thus confirms our hypothesis that the nerves would show a more severe pathology than skin with more severe inflammation. This concurs with the model of leprosy nerve damage that Scollard proposed in which he noted that inflammation occurs at every stage of leprosy nerve damage [32]. Porichha *et al* has reported on the neural pathology in nerve biopsies taken from patients with acute neuritis. Their findings show both macrophage and epithelioid cell granulomas and caseous necrosis was common in these nerves [33]. Because of the absence of non-reactional nerves we were not able to compare reactional with non-reactional nerve pathology.

The biopsies from the LL patients showed some immunological activity with 64% of biopsies having some detectable TNF-α, some (43%) TGF-β but only a few (29%) with detectable iNOS. This is compatible with the LL lesions having some inflammation but little anti-mycobacterial activity. With the panel of cytokines that we used we cannot comment on the relative Th1/Th2 profiles of cytokines produced within lesions because we were not able to stain for cytokines such as IFN-γ which are critical to making that distinction.

Twenty percent of the skin biopsies from clinically determined lesions showed either indeterminate leprosy or non-specific inflammation. This was surprising because the patients had to have at least five leprosy lesions and recruitment was done by clinicians. The presence of 13% indeterminate cases in this cohort shows that there are still many patients with early leprosy in North India. These indeterminate cases have the potential to either self heal or develop into established leprosy. Cytokines indicating ongoing immunological activity and the capacity for mycobacterial killing were detected in up to 33% of the indeterminate biopsies and this therefore parallels the clinical skin signs. Six percent of the skin biopsies showed non-specific inflammation which could be either due to the biopsy missing the critical area, a sampling error, or it could reflect a self-healing process.

One of the limitations of this study was that paraffin blocks were used for cytokine detection, thus limiting the investigation because monoclonal antibodies for detecting many cytokines cannot be used in paraffin blocks.

This study has demonstrated that cytokines were significantly associated with leprosy skin and nerve reactions and they may be of use in diagnosing and assessing difficult reaction lesions. They also indicate the complex immune-regulation in leprosy reactions which requires further investigation.
